# Efficacy and underlying mechanisms of berberine against lipid metabolic diseases: a review

**DOI:** 10.3389/fphar.2023.1283784

**Published:** 2023-11-15

**Authors:** Yajie Cai, Qiaoning Yang, Yanqiao Yu, Furong Yang, Ruina Bai, Xiaodi Fan

**Affiliations:** ^1^ Xiyuan Hospital, China Academy of Chinese Medical Sciences, Beijing, China; ^2^ National Clinical Research Center for Chinese Medicine Cardiology, Xiyuan Hospital, China Academy of Chinese Medical Sciences, Beijing, China; ^3^ NMPA Key Laboratory for Clinical Research and Evaluation of Traditional Chinese Medicine, Beijing, China; ^4^ Department of Graduate School, Beijing University of Chinese Medicine, Beijing, China; ^5^ Institute of Basic Medical Sciences, Xiyuan Hospital of China Academy of Chinese Medical Sciences, Beijing, China; ^6^ Key Laboratory of Pharmacology of Chinese Materia Medica, Beijing, China

**Keywords:** berberine, berberine metabolite, intestinal-hepatic-vascular tissue, pharmacological mechanism, lipid metabolic disease

## Abstract

Lipid-lowering therapy is an important tool for the treatment of lipid metabolic diseases, which are increasing in prevalence. However, the failure of conventional lipid-lowering drugs to achieve the desired efficacy in some patients, and the side-effects of these drug regimens, highlight the urgent need for novel lipid-lowering drugs. The liver and intestine are important in the production and removal of endogenous and exogenous lipids, respectively, and have an important impact on circulating lipid levels. Elevated circulating lipids predisposes an individual to lipid deposition in the vascular wall, affecting vascular function. Berberine (BBR) modulates liver lipid production and clearance by regulating cellular targets such as cluster of differentiation 36 (CD36), acetyl-CoA carboxylase (ACC), microsomal triglyceride transfer protein (MTTP), scavenger receptor class B type 1 (SR-BI), low-density lipoprotein receptor (LDLR), and ATP-binding cassette transporter A1 (ABCA1). It influences intestinal lipid synthesis and metabolism by modulating gut microbiota composition and metabolism. Finally, BBR maintains vascular function by targeting proteins such as endothelial nitric oxide synthase (eNOS) and lectin-like oxidized low-density lipoprotein receptor-1 (LOX-1). This paper elucidates and summarizes the pharmacological mechanisms of berberine in lipid metabolic diseases from a multi-organ (liver, intestine, and vascular system) and multi-target perspective.

## 1 Introduction

In recent years, with economic development and changes in diet, the incidence of lipid metabolic diseases, such as non-alcoholic fatty liver disease (NAFLD), atherosclerosis (AS), obesity, and hyperlipidemia, has increased, placing a significant burden on human health and the health insurance system (Loomba et al., 2021; Fan and Watanabe, 2022). Elevated lipid levels are a common feature of these diseases, and thus lipid-lowering therapies are essential for their treatment. Commonly used lipid-lowering drugs such as statins, protein convertase subtilisin/kexin type 9 (PCSK9) inhibitors, and ezetimibe have been developed based on critical targets of lipid metabolism. The widespread use of these drugs effectively lowers lipid levels and reduces lipid deposition in tissues such as blood vessels. However, adverse effects of long-term treatment, such as liver damage and rhabdomyolysis, have been widely documented, emphasizing the need to develop new lipid-lowering therapies (Liu et al., 2019).

**FIGURE 1:**
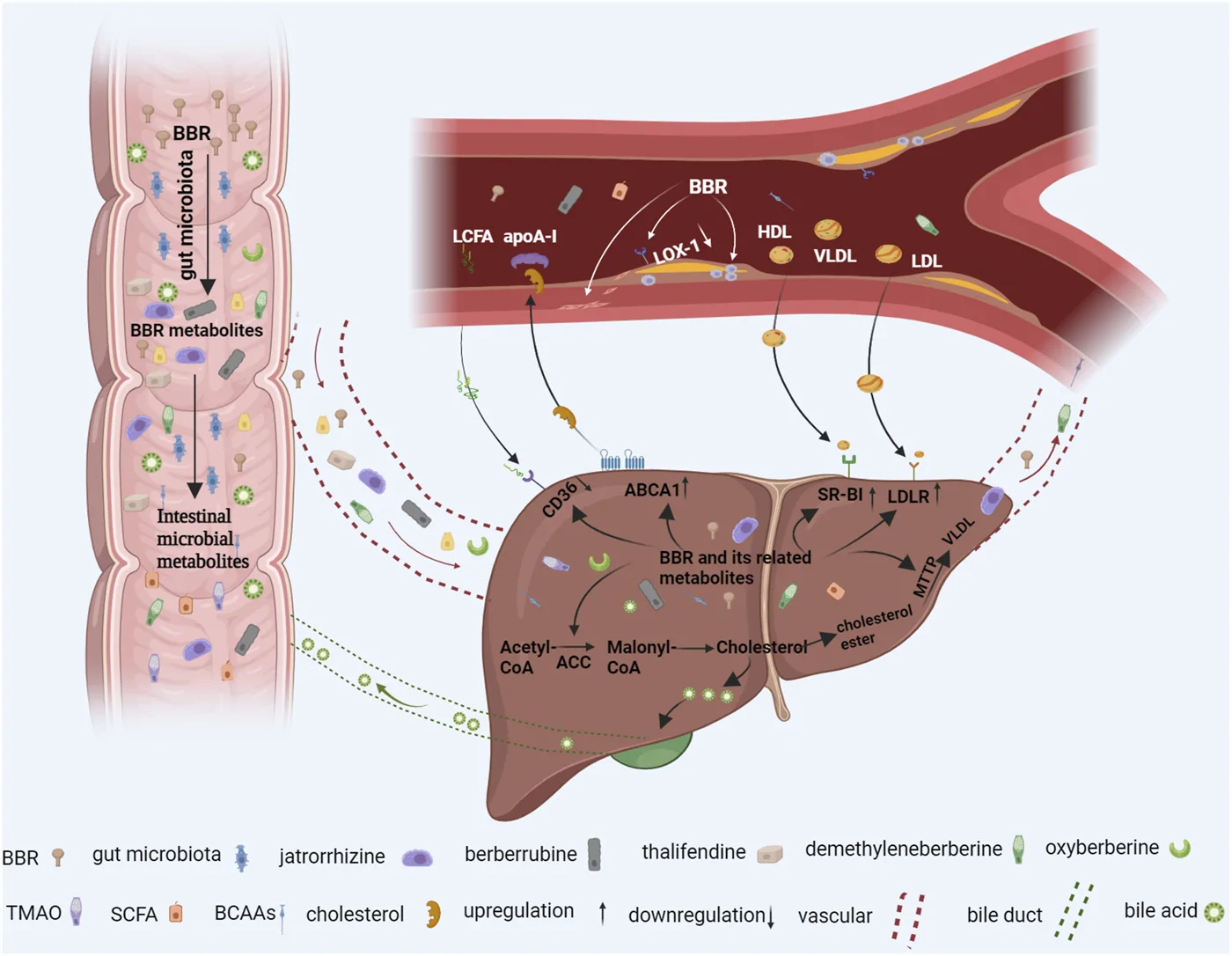
The berberine (BBR) pathway in the prevention and treatment of lipid metabolic diseases. When oral BBR enters the gut, it interacts with intestinal microorganisms. Intestinal flora convert BBR into BBR derivatives such as demethyleneberberine, jatrorrhizine, berberrubine, and thalifendine BBR can also directly regulate intestinal branched-chain amino acid (BCAA), short-chain fatty acid (SCFA), and trimethylamine-N-oxide (TMAO) production. These intestinal metabolites are absorbed into the liver through blood vessels, which reduce liver lipid production and increase circulating lipid removal by inhibiting liver cluster of differentiation 36 (CD36), acetyl-CoA carboxylase (ACC), and microsomal triglyceride transfer protein (MTTP) expression and upregulating ATP-binding cassette transporter A1 (ABCA1), scavenger receptor class B type 1 (SR-BI), bile acid, and low-density lipoprotein receptor (LDLR) expression. In addition, metabolites entering blood vessels also act directly on blood vessel walls to reduce vascular endothelial dysfunction, reduce the formation of macrophage foam cells, inhibit vascular smooth muscle cell proliferation, and, thus, alleviate atherosclerosis (AS). Additional figure abbreviations: apoA-1, apolipoprotein AI; HDL, high-density lipoprotein; LCFA, long-chain fatty acid; LDL, low-density lipoprotein; LOX-1, lectin-like oxidized low-density lipoprotein receptor-1; VLDL, very low-density lipoprotein.

Berberine (BBR) is widely used in Asian countries because of its good clinical efficacy and safety in reducing lipids (Song et al., 2020). Importantly, in contrast to drugs developed for a single target of lipid metabolism in a single organ, BBR can coordinate multiple key targets of lipid metabolism and intestinal flora in the intestine and liver to regulate lipid levels. It can also act directly on blood vessels to ameliorate endothelial dysfunction, inhibit macrophage foam cell formation, and regulate vascular smooth muscle cell (VSMC) proliferation and migration to prevent and treat AS (Rui et al., 2021) (Figure 1). The 2016 ESC/EAS Guidelines summarized evidence suggesting that BBR may reduce low-density lipoprotein cholesterol and triglyceride levels but emphasized that its efficacy for treating dyslipidemia requires further assessment in high-quality randomized clinical trials (Members et al., 2016). Separately, an International Lipid Expert Panel position paper supported the use of BBR as a lipid-lowering nutraceutical, particularly for patients with statin intolerance, mild hypercholesterolemia, or metabolic syndrome (Cicero et al., 2017). This article provides an in-depth review of recent advances in utilizing BBR to prevent and treat lipid metabolic disorders. It examines the progress from the per-spective of the liver, intestine, and blood vessels, and discusses how BBR acts on cellular targets including cluster of differentiation 36 (CD36), microsomal triglyceride transfer protein (MTTP), low-density lipoprotein receptor (LDLR), lectin-like oxidized low-density lipoprotein receptor-1 (LOX-1), acyl-CoA-cholesterol acyltransferase (ACAT), and adenosine monophosphate-activated protein kinase (AMPK), as well as gut microbiota. The aim of this review is to provide a modern scientific perspective for a holistic and systematic understanding of the mechanisms by which BBR regulates lipid lowering in multiple organs and to provide new ideas for treating lipid metabolic diseases.

## 2 Pharmacokinetics of BBR

### 2.1 Absorption, distribution, and excretion of BBR

Because intravenous BBR administration can cause serious clinical side effects, oral administration is still the main route of administration of BBR. In an animal study in rats, berberine (400 mg/kg) was administered orally in rats, Cmax was 0.260 μg/mL. AUC_0-t_ was low for the oral route of administration (Yu et al., 2022a). The poor absorption after BBR oral administration is caused by poor permeability, P-glycoprotein mediated efflux, hepatobiliary reexcretion, and self-aggregation. Absorbed BBR is rapidly and widely distributed in a variety of tissues such as the brain, intestine, stomach, pancreas, heart, kidney, and liver with the highest concentration in the liver (Tan et al., 2013). The liver and intestine, as the main sites of BBR metabolism, can metabolize BBR into a variety of derivatives through a variety of mechanisms (specifically, see section “2.2 Metabolism of BBR in the liver and intestine”). BBR is mainly excreted fecally, followed by through the urine and bile. When orally administered, BBR is majorly excreted through the feces and primarily in the original form. In contrast, BBR metabolites are mainly excreted through the urine and bile (Feng et al., 2021). Bile, as one of the pathways of BBR excretion, is also regulated by BBR-related signaling pathways. It has been found that BBR affects bile excretion by regulating the gut microbiota and acting on the farnesoid X receptor (FXR) -CYP7A1/CYP8B1 pathway (Guo et al., 2016; Wolf et al., 2021). However, due to the hepatoenteric circulation of bile acids (BAs), the amount of BBR and its derivatives excreted through bile is small.

### 2.2 Metabolism of BBR in the liver and intestine

#### 2.2.1 Hepatic metabolism of BBR

In both rats and humans, the liver plays a significant role in BBR metabolism (Kumar et al., 2015). BBR is mainly converted into berberrubine, demethyleneberberine, jatrorrhizine, and thalifendine via hepatic metabolism (Feng et al., 2021). Following oral administration, BBR metabolite levels are highest in the liver (Zhu et al., 2016). Moreover, the half-life of BBR in hepatic tissue is longer than that in other tissues (Wei et al., 2016b), suggesting that the liver may be the main site of BBR metabolism.

Cytochrome P450 enzymes (CYPs) mediate the phase I metabolic pathway of BBR in the liver (Guo et al., 2011a). Clinical studies have shown that CYP2D6 is the primary human CYP that produces BBR metabolites, followed by CYP1A2, CYP3A4, CYP2E1, and CYP2C19 (Guo et al., 2011b). Thus, quinidine and furafylline are important metabolic inhibitors of BBR as they inhibit CYP2D6 and CYP1A2 (Khoshandam et al., 2022), respectively. Li et al. showed that CYP2D6 and CYP1A2 can convert BBR to the BBR metabolite thalifendine. Thalifendine increases LDLR mRNA expression with an activity level of 26% of that of BBR (Li et al., 2011a; Tan et al., 2013). CYP2D6, CYP1A2, and CYP3A4 participate in demethyleneberberine production. In the liver, intracellular demethyleneberberine is 25.14 times more enriched than extracellular demethyleneberberine (Liu et al., 2022). After the formation of phase I metabolites of BBR, phase II metabolites of BBR may be formed by glucuronidation. UDP-glucuronosyltransferases (UGTs), particularly UGT1 and UGT2, are known to mediate glucuronidation. In a human study, UGT1A1, UGT1A3, UGT1A7, UGT1A8, UGT1A9, and UGT1A10 in the liver mediated jatrorrhizine glucuronidation (Zhou et al., 2013). Berberrubine, demethyleneberberine, and their corresponding glucuronides were also be produced via incubation with the liver S9 fraction, but at a slower pace than with liver microsomes (Liu et al., 2009).

Animal studies have shown that a large proportion of BBR (200 mg/kg) is distributed in the liver after oral administration, which may contribute to its low blood concentration. Among BBR, berberrubine, demethyleneberberine, and jatrorrhizine, demethyleneberberine has the highest distribution in the liver and jatrorrhizine has the lowest (Liu et al., 2010). The liver also affects BBR clearance. For example, liver tissue clearance of BBR is higher in normal rats than in rats with acute hepatitis. Therefore, clinical doses of BBR should be carefully controlled when using it to treat acute hepatitis (Dai et al., 2018). In addition, tandem mass spectrometry revealed that the content of BBR metabolites in human liver microsomes is significantly different from that in rat liver microsomes (Li et al., 2017). Therefore, data related to the rat liver metabolism of BBR should be carefully refer-enced when developing BBR drugs.

#### 2.2.2 Intestinal metabolism of BBR

To evaluate its intestinal first-pass metabolism, BBR was administered to rats via four distinct routes (intragastric, intraduodenal, intraportal, and intravenous). Significant differences are found in circulating BBR concentrations after intraduodenal and intravenous administration compared to intragastric administration, indicating that the intestinal first-pass effect of BBR is substantial (Liu et al., 2010). Moreover, utilizing intestinal post-mitochondrial (S9), cytosolic, and microsomal fractions, researchers have examined the intestinal metabolism of BBR (Ma et al., 2014). Five metabolites (berberrubine, demethyleneberberine, jatrorrhizine, berberrubine glucuronide, and demethyleneberberine glucuronide) were produced in the enterocyte fraction S9 and intestinal perfusates *in vitro* studies. Among them, berberrubine, demethyleneberberine glucuronide, and jatrorrhizine were the major intestinal metabolites (Liu et al., 2010).

Moreover, the intestinal flora is the largest microecosystem in the body and significantly affects material and energy metabolism. CYP51 in intestinal flora stably binds BBR and facilitates its conversion into demethylated metabolites. Adding a CYP51 inhibitor slows BBR metabolism, preventing the production of demethylated metabolites, including thalifendine and berberrubine (Zhang et al., 2021a). Furthermore, the intestinal flora can also increase circulating BBR levels by converting BBR to a more readily absorbed form, dihydroberine (DHB), via nitroreductase. Additionally, observations of the gut microbial modulation of pharmacokinetics in beagle dogs after oral administration of BBR (50 mg/kg/d) by single or multiple doses for 7 days revealed that their fecal BBR excretion levels were higher on days 3 and 7. Butyrate in the plasma and feces increased 3.1-fold and 2.7-fold, respectively, after 1 day of BBR treatment, and the number of bacteria that produce butyrate and nitroreductase was elevated after 7 days of treatment. The plasma and feces were found to contain eleven metabolites, including eight phase I and three metabolites of phase II (Feng et al., 2018b). These studies suggest that the gut and its flora are essential for BBR metabolism.

## 3 The role of BBR in regulating lipid metabolism in the liver

The liver is an essential organ for endogenous lipid production, and BBR can regulate hepatic lipid metabolism to prevent and treat lipid metabolic diseases, such as and NAFLD. The mechanisms of action of BBR include inhibiting hepatic lipid production, increasing hepatic lipid clearance, improving insulin resistance, promoting AMPK phosphorylation (p-), influencing apoptosis and autophagy in lipid cells, and regulating epigenetic modifications.

### 3.1 BBR regulates lipid production in the liver

CD36, acetyl-CoA carboxylase (ACC), and MTTP are essential regulators of lipid uptake and synthesis in the liver. BBR reduces circulating lipid levels and hepatic lipid accumulation by inhibiting these regulators.

#### 3.1.1 CD36

CD36 belongs to the scavenger receptor family, also known as fatty acid translocases, which facilitates the uptake of long-chain fatty acids (LCFAs) (Zhong et al., 2017). Hepatocyte CD36 is a crucial regulator of *de novo* hepatic lipogenesis. High fat diet (HFD)-induced liver steatosis and insulin resistance are attenuated in CD36-knockout mice (Zeng et al., 2022). Excessive accumulation of liver fat is a crucial factor in the pathogenesis of NAFLD. Therefore, suppression of CD36 to reduce liver fat uptake and synthesis may have a potential effect in preventing and treating NAFLD. Berberrubine, a main BBR metabolite, alleviates NAFLD by reducing CD36 expression Moreover, berberine maintains glucose homeostasis in HFD-fed mice by upregulating glucose transporter 2 (GLUT-2) and glycogen synthase kinase 3β (GSK3β) protein expression and inhibiting glucose 6 phosphatase (G6Pase) protein expression (Yang et al., 2022c).

Accumulating evidence has indicated that CD36 is an essential regulator of intracellular fatty acid homeostasis (Samovski et al., 2015). CD36 is involved in fatty acid oxidation (Samovski et al., 2015) and lipophagy (Li et al., 2019a) by activating AMPK, thus regulating the storage or use of fatty acids. These studies suggest that promoting fatty acid oxidation and correcting lipophagy defects by inhibiting CD36 expression in hepatocytes may be a novel strategy for treating liver fat accumulation. BBR lowers cholesterol by inhibiting bile salt hydrolase (BSH), increasing conjugated BA levels, and activating the FXR signaling pathway, among other mechanisms; this leads to reduced hepatic CD36 levels, thereby reducing both hepatic LCFA absorption and lipid accumulation (Sun et al., 2017). Therefore, BBR may play an important role in the prevention and protection against NAFLD by regulating CD36 expression.

#### 3.1.2 ACC

ACC is a crucial enzyme involved in fatty acid biosynthesis and metabolism. It has two tissue-specific isomers, ACC1 and ACC2, which catalyze the conversion of acetyl coenzyme A to malonyl coenzyme A and exhibit different distributions. A cytosolic enzyme expressed mainly in adipose tissue such as the liver, ACC1 is responsible for the rate-limiting step in LCFA biosynthesis (Munday, 2002). The rate-limiting enzyme ACC carboxylates acetyl coenzyme A into malonyl coenzyme A during adipogenesis, subsequently converting this into LCFAs and then into triglycerides (TGs) via a multi-step reaction with fatty acid synthase (FAS) (Gathercole et al., 2011). Over-synthesis of TG is a major pathogenic factor in NAFLD. Indeed, several studies have shown that ACC1 inhibitors can combat NAFLD (Wu and Huang, 2019). In HFD-induced NAFLD rats, the protein expression levels of sirtuin 3 (SIRT3), p-AMPK, and p-ACC were significantly higher in the livers of rats in the BBR treatment group, and the serum and liver lipid profiles and liver injury status improved. These data suggest that the mechanism by which BBR ameliorates HFD-induced hepatic steatosis may be related to activation of the liver SIRT3/AMPK/ACC pathway (Zhang et al., 2019b). In addition to NAFLD, BBR improves hepatic lipid metabolism in rats with alcohol-induced alcoholic fatty liver disease by reducing hepatic lipid synthesis through the inhibition of ACC, which may be related to the thyroid hormone responsive gene responsible for fatty acid synthesis (Ke et al., 2022). Single-drug therapy for fatty liver disease is typically ineffective, as it has a complex etiology and affects multiple systems (Brown et al., 2021; Zhu et al., 2021). Therefore, combining drugs with different mechanisms may enhance overall efficacy (Dufour et al., 2020; Kessoku et al., 2020). For example, the p62/nuclear factor erythroid 2-related factor 2 (NRF2)/carboxylesterase 2 (CES2) and p62/NRF2/PPARα signaling axes are restored by bicyclol, which enhances lipolysis and β-oxidation, while BBR reduces *de novo* lipogenesis by suppressing ACC and FAS expression; the combination of the two drugs has already been demonstrated to enhance their overall therapeutic effect in improving NAFLD (Li et al., 2022a).

#### 3.1.3 MTTP

NAFLD is characterized by considerable accumulation of TGs in the liver, which, under normal circumstances, are excreted from the liver as very low-density lipoprotein (VLDL). Therefore, the timely synthesis and secretion of VLDL are vital for mitigating hepatic TG deposition. The assembly and maturation of VLDL are closely related to MTTP activity (Anastasia et al., 2021). Lipids and apolipoprotein B (ApoB) are essential components of VLDL, and MTTP stabilizes ApoB expression to reduce ApoB degradation and promote lipid transfer to VLDL particles to facilitate VLDL maturation (Demignot et al., 2014). Wang et al. (Wang et al., 1999) found that inhibition of MTTP activity impedes VLDL assembly, leading to increased intrahepatic TG levels. Lomitapide is a drug developed to target the downregulation of MTTP activity. It reduces ApoB secretion but causes side effects such as increased hepatic lipid compensation and exacerbated NAFLD, thus limiting its range of application (Stefanutti, 2020). It is believed that decreased MTTP expression in NAFLD rats may decrease hepatic TG excretion in VLDL, favoring the accumulation of hepatic fat. BBR treatment reverses the abnormal HFD-induced expression of MTTP thus improving fatty liver (Chen et al., 2021). DNA methylation is one of the pathways that leads to gene expression dysregulation via interactions with environmental factors. DNA methylation levels in the MTTP promoter of NAFLD rats are elevated in the liver, and a strong negative correlation has been observed between MTTP expression and DNA methylation. BBR selectively inhibits HFD-induced MTTP methylation to partially counteract HFD-induced MTTP dysregulation, which promotes normal levels of VLDL secretion in the liver, thereby reducing hepatic fat content and alleviating NAFLD (Chang et al., 2010).

### 3.2 BBR regulates lipid clearance in the liver

Circulating high-density lipoprotein cholesterol (HDL-C) and low-density lipoprotein cholesterol (LDL-C) are taken up and cleared by hepatocytes via hepatic scavenger receptor class B type I (SR-BI) and LDLR, respectively. The liver transfers cholesterol to apolipoprotein AI (ApoAI) via the ATP-binding cassette transporter A1 (ABCA1) to form high-density lipoprotein (HDL), which is a protective factor for blood lipids to a certain extent. BBR reduces circulating and hepatic lipid levels by acting on these targets of lipid metabolism.

#### 3.2.1 SR-BI

SR-BI is a hairpin-looped structure with two short transmembrane domains, two cytoplasmic tails, and a large extracellular loop. A large cavity traverses the entire length of the SR-BI molecule, and mutagenesis of SR-BI showed that the cavity is a lipophilic tunnel through which cholesterol ester is delivered from the bound lipoprotein to the outer leaflet of the plasma membrane (Neculai et al., 2013). SR-BI is highly expressed in human and animal livers and can lower circulating cholesterol levels by taking up cholesterol from circulating HDL, VLDL, and other lipoproteins (Yang et al., 2013). In the SR-BI−/− mouse model, circulating total cholesterol (TC) levels increase 2.5-fold (Cao et al., 2018). While, overexpression of SR-BI in mouse liver can reduce the level of circulating lipid (Zhang et al., 2005). In addition, SR-BI variants in humans can lead to increased circulating cholesterol levels and an increased risk of cardiovascular disease (CVD) (May et al., 2021). Thus, regulation of SR-BI expression can influence lipid levels and AS progression.

In HFD-fed ApoE−/− mice, circulating lipid levels increased and hepatic SR-BI expression decreased. After addition of BBR, SR-BI expression was restored and the levels of circulating TC, TG, LDL-C and HDL-C decreased, delaying the progression of AS plaques and hepatic steatosis (Ma et al., 2021a). BBR may regulate SR-BI via various mechanisms. Liver X receptors (LXRs) and peroxisome proliferator-activated receptors (PPAR-α, PPAR-β/δ, and PPAR-γ) regulate human SR-BI gene expression by forming a heterodimer with the retinoid X receptor (RXR) and bind to the SR-BI promoter (Shen et al., 2018). Mitogen-activated protein kinase (MAPK) affects hepatic SR-BI expression by targeting PPAR, further altering the ability of cells to export cholesterol (Wood et al., 2011).

The mechanisms related to the regulation of lipid metabolism by BBR via hepatic SR-B1 have not been well studied. Considering that LXR, PPAR, and MAPK are known targets of BBR (Han et al., 2015; Bansod et al., 2021) and that LXR, PPAR, and MAPK have regulatory effects on hepatic SR-B1, whether BBR can affect circulating cholesterol levels by regulating hepatic SR-B1 via LXR, PPAR, and MAPK deserves further exploration.

#### 3.2.2 LDLR

Plasma LDL is mainly removed from circulation through LDLR, and LDLR deficiency increases circulating cholesterol levels and accelerates AS (Xu and Weng, 2020). Three proteins are known to bind to AU-rich element (ARE): heterologous nuclear ribonucleoprotein D (hnRNP D), hnRNP I, and KH-type splicing regulatory protein (KSRP) (Li et al., 2009a; Pan et al., 2020). These proteins interact with the LDLR 3′UTR, leading to a reduction in LDLR mRNA levels. BBR stabilizes LDLR expression by reducing the affinity between hnRNP I or KSRP and LDLR (Singh et al., 2014; Momtazi et al., 2017). Poly A-binding protein (PABP) maintains mRNA stability by protecting it against nucleophilic attack (Behm-Ansmant et al., 2007). *In vitro*, BBR indirectly enhances LDLR mRNA stability by promoting binding between PABP and the poly adenine (polyA) tail of LDLR mRNA. BBR also improves LDLR mRNA stability by targeting the polyA tail directly (Yuan et al., 2015). BBR stabilizes LDLR mRNA and enhances plasma LDL-C clearance by activating the AMPK/extracellular signal-regulated kinase (ERK) signaling pathway (Abidi et al., 2005). BBR can directly upregulate LDLR mRNA expression in hepatocytes by activating the Jun N-terminal kinase (JNK)/c-Jun signaling pathway (Fan et al., 2021), resulting in increased LDL uptake, lower circulating LDL-C levels, and alleviation of AS. These studies suggest that BBR plays a role in reducing circulating cholesterol levels by stabilizing or increasing LDLR mRNA expression.

LDLR levels are also regulated by PCSK9, which promotes LDLR degradation and increases circulating LDL-C levels by binding to LDLR (Ataei et al., 2022). BBR reduces serum LDL-C levels and delays aortic plaque formation in ApoE−/− mice by activating ERK1/2 to downregulate PCSK9 (Ma et al., 2021b). Sterol regulatory element-binding protein (SREBP) and hepatocyte nuclear factor 1α (HNF1α) control PCSK9 protein synthesis at the transcriptional level. Through ubiquitin-proteasome degradation, BBR inhibits SREBP2 and HNF1α expression. As a result, blood PCSK9 levels could be lowered via decreased transcription of its mRNA, resulting in increased LDLR mRNA expression (Cao et al., 2018; Shafabakhsh et al., 2021). Furthermore, when added to hepatocytes with statins, BBR counteracts the statin-inducted transcription of PCSK9 (Li et al., 2009b) and is therefore a potential drug for lipid regulation in combination with statins. In conclusion, BBR can not only increase LDLR expression by stabilizing LDLR mRNA, but also reduce LDLR degradation by down-regulating PCSK9, which indirectly improves LDLR expression and increases circulating cholesterol clearance.

#### 3.2.3 ABCA1

Human ABCA1 gene has been mapped to chromosome 9q31.1, spans 149 kb, and comprises 50 exons and 49 introns (Santamarina-Fojo et al., 2000). The study found that ABCA1 may transport cellular cholesterol and phospholipids to extracellular ApoAI through nine different models (Sun and Li, 2022) to generate nascent HDL particles (Ogura, 2022). Upregulation of ABCA1 promotes intracellular lipid efflux and increases circulating HDL levels to alleviate lipid metabolic diseases (Matsuo, 2022). Relative to wild-type mice, ApoE−/− mice fed an HFD exhibit reduced hepatic ABCA1 expression, whereas BBR treatment enhances the expression of ABCA1 and alleviates atherosclerotic lesions and hepatic steatosis (Ma et al., 2021a). Protein kinase Cδ (PKCδ) increases ABCA1 serine phosphorylation and reduces the rate of ABCA1 protein degradation (Weng et al., 2018). *In vitro* and *in vivo* studies demonstrate that BBR decreases hepatic cholesterol and TG levels by increasing ABCA1 protein levels through PKCδ, whereas the PKCδ inhibitor rottlerin and PKCδ siRNA completely abolish the effect of BBR on ABCA1. Furthermore, the inhibition of ABCA1 and its siRNA eliminate the ability of BBR to lower cellular cholesterol levels. These results suggest that BBR increases ABCA1 protein levels via PKCδ, thereby reducing hepatic steatosis (Liang and Wang, 2018). Therefore, ABCA1 may be an important target of BBR for the treatment of lipid metabolic diseases.

### 3.3 BBR regulates lipid metabolism through AMPK

AMPK, which plays a key role in energy metabolism, has been the focus of research on lipid-related metabolic diseases (Wang et al., 2018). When AMPK is activated, genes linked to lipogenesis are downregulated, and energy consumption is increased. As a result, lipid buildup is reduced, and liver function is enhanced.

In HFD-fed rats, the BBR- and simvastatin-treated groups showed similar decreases in LDL-C levels (−26.8% and −28.3%, respectively), and the combination of the two drugs resulted in a more pronounced decrease in LDL levels (−46.2%) (Kong et al., 2008) and slowed AS progression, potentially via AMPK activation by BBR (Cao et al., 2013), which inhibit 3-hydroxy-3-methylglutaryl-CoA reductase (HMGCR) and cholesterol biosynthesis (Song et al., 2012). AMPK can modulate lipid metabolism by acting on SREBP, a regulator of hepatic lipogenesis (Ferré et al., 2021). p-AMPK inhibits the expression of SREBP-1c via Ser372 phosphorylation and blocks SREBP-1c nuclear translocation, thus inhibiting the transcription and translation of SREBP-1c target genes such ACC1, FAS, and stearoyl CoA desaturase 1 (SCD1) (Li et al., 2011b). The role AMPK in lipid metabolism is further supported by the discovery that BBR treatment promotes hepatic lipolysis (CPT and LPL) and reduces hepatic lipid deposition in largemouth bass via AMPK/SREBP1 (Gong, 2022). In addition, the mRNA levels of two crucial cholesterol production enzymes, 3-Hydroxy-3-Methylglutaryl-CoA Synthase (HMGCS) and HMGCR, are reduced by the activation of AMPK, which is consistent with decreased levels of SREBP-2 (Li et al., 2011a). Reduced TG and cholesterol biosynthesis, owing to reduced expression of ACC1, FAS, SCD1, HMGCS, and HMGCR, improves hepatic steatosis (Li et al., 2011b). Columbamine, a BBR metabolite, also has the advantage on lowering TG levels via the activation of AMPK (Cao et al., 2013).

BBR and its derivatives can block mitochondrial respiratory chain complex I, thereby affecting levels of intracellular AMP and ADP, and activating AMPK (Sanders et al., 2007; Turner et al., 2008). Activated AMPK promotes the expression of uncoupling protein-1 (UCP1) and −2 (UCP2) in adipose tissue and increases the expression of other thermogenic genes. Fatty acids are burned off in the form of heat (Wang et al., 2011; Zhang et al., 2011; Zhang et al., 2014). At the same time, in HFD-induced NAFLD rats, BBR-induced activation of SIRT3, a crucial gene for fatty acid oxidation, alters the expression of ACC and carnitine palmitoyltransferase-1A (CPT-1A), thereby reducing hepatic steatosis (Zhang et al., 2019a). In AMPKα1−/− HepG2 cells, the stimulating effects of BBR on the activity p-AMPKα1, p-AMPKα and AMPK, and its effects on glucose and lipid metabolism, are completely suppressed (Ren et al., 2020). These studies suggest that BBR may reduce circulating fat levels by inhibiting adipogenesis and promoting fat consumption through AMPK and may be used to treat lipid metabolic diseases.

### 3.4 BBR affects hepatic lipid metabolism through other pathways

#### 3.4.1 Mitochondria

The mitochondria are an important location for energy (including lipid) metabolism in cells, and are composed of an outer membrane, intermembrane space, inner membrane, and matrix. Studies have shown that excessive lipid accumulation in hepatocytes during NAFLD leads to abnormal fatty acid oxidation, increased mitochondrial reactive oxygen species production, and abnormal mitochondrial membrane lipids and proteins (Prasun et al., 2021). Mitochondria-associated ER membranes (MAMs) are lipid raft-like domains closely associated with mitochondria, which are located between mitochondria and the endoplasmic reticulum. MAMs not only structurally connect the endoplasmic reticulum and mitochondria, but are also rich in a variety of functional proteins related to lipid metabolism such as acyl-coA:cholesterol acyltransferase-1 (ACAT1/SOAT1), acyl-CoA:diacylglycerol acyltransferase 2 (DGAT2) (Ma et al., 2021b), and phosphatidylethanolamine N-methyltransferase (PEMT) (Zhao et al., 2009). Administration of BBR to rats fed a high-fat diet reduced the expression of DGAT in liver tissues and the level of circulating blood lipids (Zhu et al., 2017). *In vitro* experiments have shown that BBR can inhibit ATP synthesis, enhance glycolysis, and promote glucose metabolism by inhibiting the mitochondrial respiratory chain complex I (Xu et al., 2014). Animal experiments have shown that BBR upregulates the mitochondrial content in brown and white adipocytes in diabetic mice, stimulates UCP1 -mediated thermogenesis, and accelerates fat catabolism. These mechanisms may be related to AMPK signaling (Zhang et al., 2014). In addition, Sirtuin 3 (SIRT3), a deacetylase mainly located in mitochondria, is a key regulator of mitochondrial function. SIRT3 is highly expressed in metabolically active tissues (such as the liver). Mice fed a high-fat diet for sustained periods exhibit decreased Sirt3 expression, resulting in mitochondrial dysfunction and over-acetylation of proteins in the liver. These changes can increase the risk of aging-related diseases such as NAFLD and obesity (Choudhury et al., 2011). Potential mechanisms underlying these findings include the downregulation of ERK-CREB-Bnip3 and inhibition of the mitochondrial autophagy pathway (Li et al., 2018a). BBR also promotes fatty acid β oxidation through SIRT3-LCAD, thereby slowing the progression of NAFLD (Xu et al., 2019). By activating SIRT3, BBR can also improve adipose tissue remodeling, downregulate the expression of TNF-α and NF-kB, which play an anti-hyperlipidemic and anti-hyperglycemic role (Li et al., 2022b). Therefore, by regulating mitochondria, MAMs, and their related proteins is one of the important ways for BBR to exert lipid-lowering effects.

#### 3.4.2 Autophagy

Lipid droplets are separated by autophagy and sent to hepatocyte lysosomes for degradation, enabling the breakdown and excretion of lipids such as TG and cholesterol (Zhang et al., 2018). The Akt/mammalian target of rapamycin (mTOR) axis is the main signaling pathway that regulates autophagy (Jung et al., 2010). Autophagy activation by mTOR inhibitors promotes lipid degradation (Lin et al., 2013). BBR slows NAFLD progression by inhibiting ERK/mTOR-induced autophagy, thereby improving hepatic lipid accumulation *in vitro* and *in vivo* (He et al., 2017). BBR enhances autophagy by preventing cyclooxygenase-2 (COX-2)-mediated prostaglandin formation, thereby reducing p-AKT and p-mTOR levels (Sun et al., 2018a; Gendy et al., 2022). In ApoE−/− mice fed a high-fat diet, BBR regulates autophagy, reduces lipid levels, and antagonizes carotid lipid accumulation by modulating the phosphatidylinositol 3-kinase (PI3K)/AKT/mTOR signaling pathway (Song and Chen, 2021). Moreover, SIRT1 is essential for BBR to potentiate autophagy and inhibit lipid storage in the mouse liver in response to fasting. BBR stimulates SIRT1 deacetylation and induces autophagy in an autophagy-related protein 5 (ATG5)-dependent manner (Sun et al., 2018b). Therefore, promoting autophagy is a key method by which BBR reduces circulating lipid levels.

#### 3.4.3 Insulin resistance

Increasing evidence suggests that insulin resistance and lipid metabolism are tightly connected as tissue lipid accumulation could lead to severe insulin resistance (Yang et al., 2018). In addition, the elevation of insulin levels after a meal signal the liver to adjust lipid metabolism (Titchenell et al., 2017). Moreover, lipid metabolic diseases, such as obesity, hyperlipidemia, and AS, are often associated with insulin resistance (Lee et al., 2022). BBR can improve insulin resistance (Yan et al., 2015) by upregulating insulin receptor substrate-2 (IRS-2) (Xing et al., 2011) and down regulating UCP2 at the mRNA and protein levels along with a decrease in TC, TG, and LDL-C and an increase in HDL-C in HFD-induced NAFLD model rats (Zhao et al., 2017).

#### 3.4.4 Epigenetic modification

Long noncoding RNA (lncRNA) and microRNA (miRNA) are important regulators of lipid homeostasis. BBR therapy reverses the expression of numerous genes, including 881 mRNAs and 538 lncRNAs, in steatotic liver (Yuan et al., 2015). In BBR-treated immortalized hepatocyte cell lines MIHA and HepG2, upregulation of miR-373 levels is observed, which in turn inhibits the AKT/mTOR/ribosomal S6 kinase (S6K) signaling pathway associated with steatosis in hepatocytes, thereby reducing abnormal lipid deposition in the liver (Li et al., 2018b). In addition, BBR therapy prevents type 2 diabetic mice from developing hepatic gluconeogenesis and lipid metabolism abnormalities, by lowering miR122 (Wei et al., 2016a). Moreover, BBR affects the expression of lncRNAs associated with the rapamycin, MAPK, and apoptosis pathways in the plasma of patients with stable coronary artery disease, which may be involved in coronary heart disease prognosis (Han et al., 2022). DNA demethylation and histone acetylation are also involved in BBR-mediated lipid regulation. L-type pyruvate kinase (L-PK) is the third rate-limiting enzyme in glycolysis and is closely associated with NAFLD and diabetes. BBR treatment restores the expression of L-PK by demethylating the L-PK promoter and increasing the acetylation levels of histones H3 and H4 around L-PK and could therefore be used to treat NAFLD (Zhang et al., 2015). BBR treatment reduces hepatic lipid deposition by reversing abnormal epigenetic modifications, such as DNA modification, histone modification, and noncoding RNA regulation, thereby maintaining normal hepatic lipid metabolism.

## 4 The role of BBR in regulating lipid metabolism in the intestine

**FIGURE 2:**
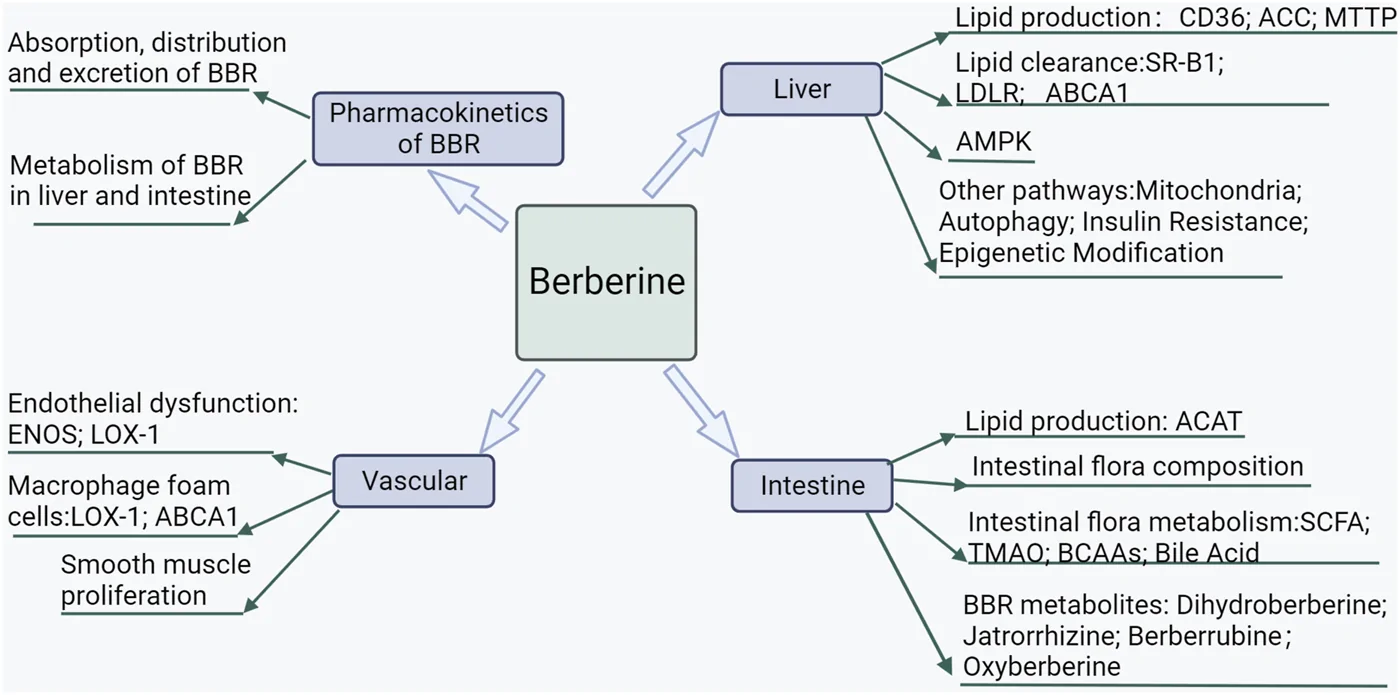
Mechanisms of BBR prevention and treatment of lipid metabolic diseases.

**FIGURE 3:**
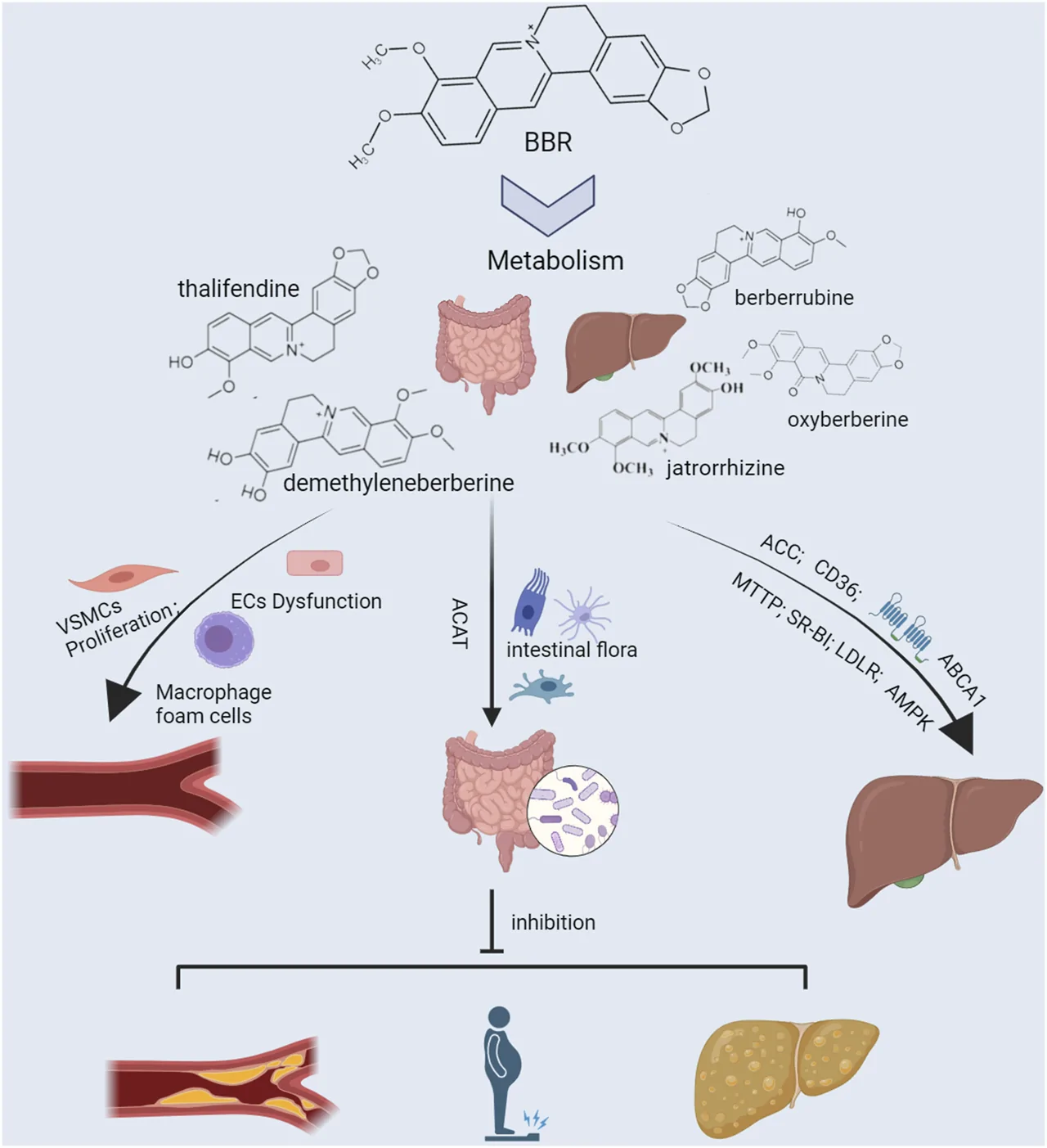
Efficacy and underlying mechanisms of berberine against lipid metabolic diseases. The liver and intestine are important organs for endogenous and exogenous lipid production, respectively, and regulate the body’s lipid levels. Cluster of differentiation 36 (CD36), acetyl-CoA carboxylase (ACC), and microsomal triglyceride transfer protein (MTTP) are the liver targets for lipid production. At the same time, ATP-binding cassette transporter A1 (ABCA1), scavenger receptor class B type 1 (SR-BI), and low-density lipoprotein receptor (LDLR) are the liver targets for lipid clearance. Risk factors such as irrational diet and disease promote lipogenesis and inhibit lipid clearance by up-regulating CD36, ACC, and MTTP and down-regulating SR-BI, LDLR, and ABCA1, elevating circulating lipid levels and exacerbating hepatic fat accumulation. Intestinal flora is an important micro-ecosystem of the intestine, which is greatly influenced by diet. High-fat diet aggravates the body’s lipid burden by altering the intestinal flora’s composition and its metabolites’ production. Elevated circulating lipid levels lead to vascular endothelial dysfunction, macrophage foam cell formation, and vascular smooth muscle cell hyperproliferation, accelerating the formation of atherosclerosis. Berberine works against lipid metabolic diseases by synergistically regulating multiple lipid metabolism-related targets in the liver, gut, and blood vessels.

Like the endogenous lipid-producing organ, the liver, the intestine is an important organ for exogenous lipid production and makes an important contribution to the body’s lipid levels (Figures 2, 3). BBR can prevent and treat lipid metabolic diseases by directly regulating intestinal lipid metabolism and interacting with intestinal flora.

### 4.1 BBR affects lipid production in the gut

Compared to the many adverse effects of ACAT1 deficiency, such as cholesterol accumulation, macrophage death, and destabilization of cell membrane function, ACAT2 deficiency protects against AS (Rudel et al., 2005). ACAT2 promotes the conversion of cholesterol into cholesteryl esters (CEs), which in turn synthesize lipoproteins and affect lipid levels. Furthermore, ACAT2 affects intestinal cholesterol absorption, which is significantly reduced in mice lacking ACAT2 (Temel et al., 2005).

Free cholesterol is incorporated into mixed micelles in the intestinal lumen after fat digestion and de-esterification, before being delivered to the small intestine’s absorptive epithelium. This step determines the amount of cholesterol available for uptake on the apical side of the enterocytes. ACAT2 converts free cholesterol taken up by intestinal cells into CEs in the intestine and further assembles CEs into chylomicrons (CMs). In HFD-fed rats, BBR treatment reduces the level of circulating total and non-HDL cholesterol. *In vitro* studies reveal that BBR treatment reduces the monolayer permeability of Caco-2 cells and their uptake and esterification of cholesterol, the mechanism of which might be related to the inhibition of ACAT2 gene and protein expression in Caco-2 cells by BBR (Wang et al., 2014). This study suggests that BBR may affect blood lipid levels by inhibiting intestinal ACAT2 and may be used to combat lipid metabolic disorders. Studies on the relevant targets of BBR in regulating intestinal lipid metabolism are currently scarce and need to be conducted in the future.

### 4.2 BBR affects lipid metabolism by affecting intestinal flora composition

Because of the low oral bioavailability of BBR and its direct antibacterial effect (Huang et al., 2020), it can alter intestinal flora composition (Hu et al., 2022b). In HFD-induced atherosclerotic mice, BBR treatment exerts a beneficial effect on reducing circulating lipid levels (Wu et al., 2020) by increasing the abundance of *Akkermansia, Firmicutes,* and *Verrucomicrobia* (Zhu et al., 2018) and decreasing the abundance of *Bacteroidetes* and *Proteobacteria* in the mouse intestinal flora (Shi et al., 2018). In HFD-induced NAFLD rats, BBR reduces body weight and TC, TG, and LDL-C levels. This improved phenotype is associated with an increase in intestinal *Bacteroidetes* and P*roteobacteria* and a decrease in *Firmicutes* and *Cyanobacteria* in rats treated with BBR (Wang et al., 2020b).

A clinical study found that circulating cholesterol levels decrease significantly after BBR treatment, with little change in TG levels. Simultaneously, suppression of *Blautia* in the gut flora eliminates the cholesterol-lowering effect of BBR, suggesting that the lipid-lowering effect of BBR is closely related to its modulation of the gut microbiota. Interestingly, baseline levels of *Blautia* accurately predict the anti-hypercholesterolemic efficiency of BBR in subsequent treatments (Wu et al., 2022). Microbiome analysis indicates that *Blautia* is closely associated with BBR lipid-modulating activities and that BBR selectively promotes *Blautia producta* (Wu et al., 2022). Further studies found that *B. producta* significantly increases LDLR expression in the liver and contributes to the role of BBR in the production of butyrate and the inhibition of BSH, thereby reducing obesity and alleviating hyperlipidemia in mice (Yang et al., 2022a). The intestinal microbiota, especially *B. producta*, may contribute to the BBR-directed prevention and treatment of lipid metabolic diseases (Yang et al., 2022b).

In addition to intestinal bacteria, intestinal fungi play an essential role in BBR-mediated regulation of lipid metabolism. *Tilletia bornmuelleri* and *Tilletia bromi* content increase after BBR treatment, reducing serum lipids and inhibiting hepatic lipid accumulation in HFD-fed mice (Yang et al., 2022). In addition, intestinal BBR levels are higher in hyperlipidemic rats than in normal rats, and BBR bioavailability is reduced after the administration of antibiotics. This suggests that the higher circulating BBR levels in hyperlipidemic rats may be related to their higher intestinal microbial population (Zhang et al., 2021a). However, another human study found a negative correlation between circulating BBR and gut microbial counts (Alolga et al., 2016). This discrepancy between studies may be due to differences in the study subjects; therefore, more research and clinical trials are needed.

In conclusion, BBR can alleviate HFD-induced lipid metabolic disorders by modulating intestinal flora composition.

### 4.3 BBR affects lipid metabolism by affecting intestinal flora metabolism

BBR interacts with intestinal microorganisms to derive a variety of metabolites, such as short-chain fatty acids (SCFAs), trimethylamine-N-oxide (TMAO), and branched-chain amino acids (BCAAs) (Fang et al., 2022). These derivatives and the signaling pathways they influence have a large impact on lipid levels and, as such, may be used to treat lipid metabolic diseases.

#### 4.3.1 SCFAs

SCFAs are carboxylic acids with two to six carbon atoms (Ohira et al., 2017). The intestinal flora syn-thesizes SCFAs, such as butyric, acetic, and propionic acids, by fermenting undigested dietary fiber (Li et al., 2022). Many studies have suggested that these SCFAs delay AS progression (Hu et al., 2022a). In HFD-fed ApoE−/− mice, the addition of propionic acid increases circulating interleukin (IL)-10 and inhibits the expression of the intestinal cholesterol transporter Niemann-Pick C1-like 1 (NPC1L1), reducing circulating cholesterol levels and alleviating AS (Haghikia et al., 2022). The addition of butyric acid downregulates the expression of Npc1l1 in intestinal cells and upregulates the mRNA levels of ATP-binding cassette transporters G5 and G8 (ABCG5 and ABCG8), thereby reducing lipid intake, increasing lipid efflux in intestinal cells, and protecting ApoE−/− mice from HFD-induced AS (Chen et al., 2018).

Both *in vitro* and *in vivo* experiments have shown that BBR increases intestinal butyrate production, thereby reducing circulating lipid levels. Moreover, intraperitoneal BBR administration does not increase butyrate levels but reduces lipid and glucose levels. This suggests that BBR reduces hyperlipidemia in two ways, through the direct effect of circulating BBR and its indirect action through intestinal microbiota (Wang et al., 2017b). BBR modifies the SCFA biosynthesis-related enzymes to control SCFA expression. (Yue et al., 2019a). found that BBR treatment significantly increased intestinal butyric acid levels by promoting the expression and activity of butyryl-CoA:acetate-CoA transferase (BUT). When BBR is present, the expression of BUT and two butyrate synthesis precursors (crotonyl-CoA and butyryl-CoA) is elevated, thereby reducing blood lipid levels (Wang et al., 2017c). BBR also regulates SCFA levels by regulating the abundance of SCFA-producing bacteria (Zhang et al., 2019a). The proportions of SCFA-producing bacteria in the gut microbiota, including of *Blautia, Bacteroidales*, and *Roseburia*, are elevated following BBR treatment (Jia et al., 2019b). Another study revealed that the SCFA producers *Butyricimonas, Eubacterium*, and *Clostridium* were significantly enriched with BBR treatment, indicating that SCFA-producing bacteria probably play a key role in the effectiveness of BBR (Mukherjee et al., 2020).

#### 4.3.2 TMAO

Trimethylamine (TMA) is synthesized from undigested choline, carnitine, and betaine obtained from food by enzymes produced by the gut bacteria (Velasquez et al., 2016). Intestinal TMA absorbed into the blood is further transformed into its pro-atherogenic form, TMAO, by flavin monooxygenase family members, such as flavin-containing monooxygenases (FMO3), in the liver. TMAO can promote AS by interfering with reverse cholesterol transport and promoting inflammation and thrombosis, among other mechanisms (Poli, 2020).

Several *in vitro* and *ex vivo* experiments have suggested that BBR may act, at least in part, by reducing TMAO during AS treatment. Decreased serum inflammatory factor expression in HFD-induced mouse models after BBR treatment might be related to the downregulation of the FMO3-TMAO pathway (Shi et al., 2018). BBR can also reduce circulating TMA and TMAO levels by downregulating enzymes associated with TMA production (Wu et al., 2020) and genes associated with TMA production (Li et al., 2021a). BBR treatment reduces intestinal TMAO biosynthesis and interrupts hamster vascular plaque formation by interacting with the choline-trimethylamine lyase (CutC) and FMO in the gut microbiota of HFD-fed hamsters. In a subsequent clinical study, patients in the BBR group were observed to have lower plaque scores and reduced circulating and fecal TMA and TMAO levels than those in the rosuvastatin plus aspirin group, suggesting that BBR might treat AS through the choline-TMA-TMAO production pathway (Ma et al., 2022). Furthermore, BBR administration reshapes the structure of rat intestinal microbiota, increasing the levels of *Lactobacillus spp*. and decreasing the levels of CutC and TMAO, leading to the attenuation of the TMAO-induced phosphorylation of ERK1/2 and JNK in platelets and reduced platelet reactivity to collagen and risk of atherothrombosis (Xie et al., 2021). Wang et al. (Wang et al., 2022) reported that BBR alleviates Ang II-induced vascular dysfunction and pathological remodeling in hypertensive mice by inhibiting FMO3 expression and TMA/TMAO production. In summary, the vasoprotective effect of BBR treatment through the inhibition of TMAO may delay AS progression.

#### 4.3.3 BCAAs

BCAAs are essential amino acids mainly obtained from food. Elevated BCAA levels are strongly connected with lipid metabolic disorders such as hyperlipidemia, obesity, and NAFLD (Cuomo et al., 2022). A case-control study found that dietary BCAAs are positively associated with circulating TC and LDL-C levels, thereby increasing the risk of dyslipidemia (Yu et al., 2022). Numerous bacterial taxa, including *Bacteroides vulgatus*, the *Bacil-lus–Lactobacillus–Streptococcus* group, *Prevotella copri*, and *Proteobacteria*, participate in BCAA synthesis (Pedersen et al., 2016). BBR regulates BCAA synthesis, probably by inhibiting microbial BCAA production. For example, treatment with BBR reduces serum TC, HDL, TG, fasting plasma glucose (FPG), and insulin resistance levels in obese mice, which may be associated with a reduction in the relative abundance of BCAA-producing bacteria, such as Clostridiaceae and *Prevotellaceae*, in the gut of these mice (Yue et al., 2019b). Abnormal mTOR activation is linked to a number of illnesses, including ischemic diseases (Hua et al., 2019); BCAAs, particularly leucine, activate the mTOR pathway (Yoon, 2016). The inhibitory effect of BBR on BCAAs may therefore play a role in reducing the risk of developing ischemic diseases via mTOR. In summary, in treating lipid metabolic disorders, it is crucial to restrict the proliferation of BCAA-producing bacteria and lower BCAA levels.

#### 4.3.4 BA

Intestinal flora can convert primary BAs to secondary BAs through decoupling and dehydroxylation reactions (Long et al., 2017). It was found that after the addition of BBR, the abundances of *Akkermansia* spp.*, Verrucomicrobia*, and *Firmicutes* increased, while those of *Proteobacteria* and *Bacteroidetes* decreased (Tian et al., 2019). Furthermore, the proportion of total BAs and primary BAs (such as cholic acid (CA) and chenodeoxycholic acid (CDCA)) in serum increased, while the proportion of secondary BAs (such as deoxycholic acid (DCA) and lithocholic acid (LCA)) was reduced. These findings were associated with increasing BBR concentrations in a dose-dependent manner (Guo et al., 2016). These changes in the BA pool can affect body function by regulating BA receptors in the gut (Martinot et al., 2017). The BA receptors mainly consist of G protein-coupled BA receptor 1 (TGR5) and nuclear receptors such as FXR (Fiorucci et al., 2018). Various BA receptors bind to different types of BAs with different affinities. For example, DCA and LCA have a high affinity for TGR5, while CDCA and CA have a high affinity for FXR. Upon BBR treatment, increased CA and CDCA activate FXR (Wolf et al., 2021). FXR plays a central role in regulating BA synthesis. FXR regulates the expression of rate-limiting enzymes (such as CYP7A1 and CYP8B1) in BA synthesis by inducing negative nuclear receptor small heterodimer partner (SHP) (Gómez-Ambrosi et al., 2017). At the same time, BBR can increase the excretion of BA and decrease the level of circulating and liver cholesterol by directly up-regulating the expression of CYP7A1 and CYP8B1 in mice (Guo et al., 2016). In addition, activated FXR can downregulate the expression of adipogenic genes, including SREGBP-1C, FAS, and acetyl-CoA carboxylase through the FXR/SHP pathway in mice, thus playing a role in the prevention and treatment of lipid metabolic diseases (Watanabe et al., 2004).

### 4.4 Intestinal flora regulate the production of BBR metabolites to affect lipid metabolism

Food and medication can be biotransformed by the gut microbiota into secondary metabolites (Wang et al., 2017a). Modern technologies show that the gut bacteria can convert BBR into DHB, berberrubine, demethyleneberberine, jatrorrhizine, and thalifendine (Habtemariam, 2020). In addition to having pharmacological effects similar to those of BBR, these metabolites also have their own characteristics and advantages and contribute significantly to the hypolipidemic effect.

#### 4.4.1 Dihydroberberine

The intestinal absorption of BBR is aided by the nitroreductase (NR) activity of the intestinal bacteria (Wang et al., 2017b). Intestinal NR catalyzes the conversion of BBR to DHB (Tan et al., 2019). Compared to BBR, DHB has a higher rate of intestinal absorption (Feng et al., 2015; Pan et al., 2019). The gut microbial metabolite DHB, which is produced from BBR, may be critical for explaining the absorption of BBR in the gut. Two procedures are involved in the circulatory sys-tem’s uptake of DHB from the intestine: first, the conversion of BBR to DHB, catalyzed by the enzymes of the intestinal microbiota, is uptaked by the intestinal epithelial cells, and second, the subsequent absorption of DHB by the intestinal epithelial cells is further oxidized to BBR, which is absorbed into the circulatory system (Feng et al., 2015). DHB is converted to BBR via a nonenzymatic process. NR plays an important role in the BBR-DHB-BBR conversion process, with enhanced BBR uptake being the basis for promoting subsequent lipid-regulating effects. In a randomized controlled crossover trial, after oral administration of 500 mg of BBR or 100 and 200 mg DHB to study subjects, the peak concentration was found to be higher in the DHB group than in the oral-administration BBR group (Moon et al., 2021). Liu et al. found that DHB improves the lipid profile of hyperlipidemic rats by increasing LDL-R expression and decreasing PCSK-9 expression in liver tissues (Liu et al., 2015b). Given its good intestinal absorption rate and lipid-lowering efficacy, DHB can potentially be used to treat lipid metabolic diseases.

#### 4.4.2 Jatrorrhizine

The gut microbiota can break down the five-membered dioxymethylene ring in BBR to create jatrorrhizine (Wang et al., 2017c). Jatrorrhizine is safer than BBR as a microbial reduction product. The lethal dose 50 (LD50) of jatrorrhizine in mice is approximately 5,500 mg/kg, whereas the LD50 of BBR is 763 mg/kg. Additionally, jatrorrhizine reduces circulating cholesterol levels by upregulating LDLR and CYP7A1 mRNA and protein expression (Wu et al., 2014). Yang et al. found that jatrorrhizine downregulates mRNA expression of SREBP-1c and FAS in the liver of hyperlipidemic mice to inhibit adipogenesis and upregulates PPAR-α and CPT1A mRNA expression to enhance lipid oxidation and improve hyperlipidemia (Yang et al., 2016). In obese mice, jatrorrhizine treatment reduces body weight and serum lipid levels and improves hepatic lipid metabolism via the IRS1/PI3K/AKT signaling pathway (He et al., 2022). In addition, jatrorrhizine treatment increases endothelial nitric oxide (NO) release; normalizes plasma lipid levels in diabetic and obese mice; and improves glucose sensitivity, fatty liver, and morphological changes, possibly related to its ability to inhibit ER and oxidative stress by enhancing the Akt/endothelial nitric oxide synthase (eNOS) pathway and NO bioavailability (Zhou et al., 2022). These studies suggest that jatrorrhizine, an intestinal microbial metabolite of BBR, may improve circulatory and hepatic lipid levels through multiple pathways.

#### 4.4.3 Berberrubine

The CYP51 enzyme in the intestinal microbiota converts BBR into demethylated metabolites such as berberrubine (Zhang et al., 2021b). Both BBR and berberrubine treatments significantly upregulate the expression of proteins associated with lipolysis (ALGL) and fatty acid β-oxidation (CPT-1 and PPAR-α) while significantly decreasing the expression of proteins associated with *de novo* adipogenesis (ACC1 and FAS) and fatty acid translation (CD36). In addition, compared with BBR, berberrubine maintains glucose homeostasis in HFD-fed mice via glucose transporter 2 (GLUT2), glycogen synthase kinase 3 (GSK3), and glucose-6-phosphatase (G6Pase) via a mechanism that may be related to the FXR signaling pathway (Sun et al., 2021). Berberrubine also acts as a cholesterol-lowering agent in human hepatoma HepG2 cells by inhibiting PCSK9 expression and upregulating LDLR expression via the ERK signaling pathway (Cao et al., 2019). Additionally, berberrubine outperforms BBR in terms of absolute bioavailability and fat solubility (Liu et al., 2016). In summary, berberrubine, a demethylated metabolite of BBR, offers more benefits than BBR, which may explain the lipid-regulating activity of BBR.

#### 4.4.4 Oxyberberine

The gut microbiota can convert BBR to oxyberberine (OBB) through oxidation, and this transformation is significantly inhibited after oral antibiotics (Li et al., 2020). When BBR was metabolized to its oxidized derivative OBB, the structure of C-8 quaternary ammonium turned to more active lactam ring, and the lipophilicity would be enhanced and easier to be absorbed through biofilm, which was beneficial to enhance its biological activity (Singh et al., 2016). In addition, OBB exhibits more favorable safety profile as compared to BBR, with LD50 value above 5,000 mg/kg in mice (the LD50 value of BBR was 713.57 mg/kg) (Li et al., 2019b). OBB can inhibit inflammation and maintain normal intestinal barrier function by inhibiting TLR4-MyD88-NF-κB signaling (Li et al., 2020). In the streptozotocin (STZ)-induced diabetic rat model, the addition of OBB significantly upregulated the expression of the Nrf2 and PI3K/Akt signaling pathways in the model group. OBB also had good hypoglycemic and protective effects on pancreatic β cells, which was superior to that of BBR at an equivalent dose (Dou et al., 2021). Another study found that OBB significantly inhibited abnormal phosphorylation of IRS in rat models of NAFLD (induced via high-fat diets), and improved hepatic insulin signaling. OBB can also reduce chronic adipose tissue inflammation by inhibiting WAT expansion, reducing macrophage migration, and promoting M2-macrophage phenotypic transformation, thus playing an anti-NAFLD role. These findings may be related to AMPK activation (Li et al., 2021b). In view of the superior effect of OBB in improving glucose and lipid metabolism, it may be a potential therapeutic option for the treatment of lipid metabolic diseases in the future.

## 5 Effect of BBR on blood vessels

Elevated lipid levels can cause endothelial cell (EC) dysfunction, foam cell formation, and VSMC proliferation, ultimately inducing the development of AS. In contrast, BBR alleviates endothelial dysfunction and inhibits macrophage foam cell formation and VSMC over-proliferation by reducing dyslipidemia (Figure 1).

### 5.1 BBR alleviates endothelial dysfunction

Cardiovascular events can be predicted based on endothelial dysfunction, which also causes AS (Xu et al., 2021). NO is a key regulator of endothelial function (Cyr et al., 2020), and hyperlipidemia not only reduces circulating NO levels by downregulating eNOS expression but also affects related pathways through LOX-1 entry into ECs, leading to endothelial dysfunction. In contrast, BBR improves endothelial function by promoting eNOS and inhibiting LOX-1 expression.

#### 5.1.1 eNOS

eNOS is the main weapon of ECs against vascular diseases, and the production of NO is essential for maintaining normal endothelial function (Benincasa et al., 2022). Hyperlipidemia can reduce NO production and exacerbate endothelial damage by downregulating eNOS through signaling pathways, such as HMGB1/TLR4/Caveolin-1 (Gliozzi et al., 2019), NRF2 (Abu-Saleh et al., 2021), and AMPK/PI3K/AkteNOS (García-Prieto et al., 2015). In turn, the damaged vascular endothelium reduces NO production and exacerbates endothelial dysfunction (Wu et al., 2019).

A previous study showed that BBR treatment reduces plasma TG levels, increases eNOS mRNA and protein expression and serum NO levels, improves endothelium-dependent vasorelaxation in the aorta, and restores endothelial dysfunction in diabetic rats (Wang et al., 2009b). In a palmitate-induced dysfunction model of human umbilical vein endothelial cells (HUVECs), the addition of BBR raised eNOS and NO levels, ameliorating palmitate-induced endothelial dysfunction, and further experiments revealed that this modulatory effect of BBR might be due to AMPK activation (Zhang et al., 2013). In addition, endothelial dysfunction is a key event linking obesity, diabetes, and CVDs. In a model of hyperglycemia-induced endothelial injury, BBR concentration-dependently enhanced eNOS phosphorylation and promoted eNOS binding to heat shock protein 90 (HSP90). This led to increased NO production, which in turn caused endothelium-dependent vasodilation and alleviated high glucose-mediated endothelial dysfunction (Wang et al., 2009a). Despite current controversies, innovative findings have shown that endothelial progenitor cells (EPCs) may be used in cell therapy for cardiovascular repair (Bianconi et al., 2018). BBR mobilizes EPCs by increasing plasma NO concentrations (Xu et al., 2009), increasing the engagement of EPCs and, consequently, the elasticity of small arteries (Xu et al., 2008). These findings suggest that BBR improves endothelial function by increasing NO production through the upregulation of eNOS expression.

#### 5.1.2 LOX-1

Circulating ox-LDL enters the vascular endothelium via LOX-1. This leads to increased intracellular reactive oxygen species (ROS) production and induces mitochondrial dysfunction by increasing NADPH oxidase (Liang et al., 2018) and myeloperoxidase (Liu et al., 2015a), and decreasing NRF2 (Huang et al., 2013). This promotes EC senescence (Bian et al., 2020) and accelerates AS progression (Jiang et al., 2020). As such, LOX-1−/− mice exhibit slow AS progression (Mehta et al., 2007).

Induction of HUVECs with ox-LDL upregulates LOX-1 expression and thus increases the uptake of ox-LDL by Ecs (Guo et al., 2022), resulting in abnormal proliferation and dysfunction of vascular ECs, possibly associated with the activation of the PI3K/Akt, ERK1/2, and MAPK signaling pathways. The subsequent addition of BBR reduces the expression of pAkt, pERK1/2, and p38MAPK, downregulates LOX-1 expression, and significantly inhibits the excessive proliferation of HUVECs, indicating that BBR is a protective agent against vascular endothelial dysfunction (Xu et al., 2017). BBR has also been shown to improve endothelial function in ApoE−/− mice by modulating mitochondrial dysfunction and targeting ApoAI to reduce plasma ox-LDL (Tan et al., 2020) levels. Further exposure of HUVEC to ox-LDL or tumor necrosis factor-alpha (TNFα) for 24 h signifi-cantly increases LOX-1 expression, which is downregulated by the addition of BBR or lovastatin. However, only treatment with BBR reduces TNFα-induced expression of vascular cell adhesion molecule-1 (VCAM-1) and intercellular adhesion molecule-1 (ICAM-1) associated with endothelial dysfunction, possibly through phosphorylation of MAPK/ERK1/2, compared to treatment with lovastatin (Caliceti et al., 2017). Therefore, it has been suggested that BBR can improve endothelial dysfunction in a multifaceted manner compared with lovastatin.

### 5.2 BBR inhibits lipid inflow and promotes lipid outflow in macrophages

Macrophage foam cells are abundant in AS plaques. BBR inhibits lipid uptake by macrophages through the downregulation of LOX-1 and promotes intracellular lipid efflux through the upregulation of ABCA1/G1, thereby reducing intracellular lipid deposition and macrophage foam cell formation and slowing AS progression.

#### 5.2.1 LOX-1

LOX-1 is also expressed in macrophages and promotes their conversion into foam cells, thereby accelerating macrophage senescence and AS progression (Akhmedov et al., 2021). Ox-LDL induces macrophage senescence by increasing lipid accumulation in macrophages and upregulating senescence-associated proteins, such as p53, p21, and p16 (Ahmad and Leake, 2019; Jia et al., 2019a). The use of an anti-LOX-1 antibody significantly reduces the uptake of ox-LDL by macro-phages and slows AS progression (Dandapat et al., 2007). In ox-LDL-induced human-derived macrophages, ox-LDL significantly increases LOX-1 expression; in contrast, BBR treatment reduces foam cell formation in a dose- and time-dependent manner and inhibits LOX-1 expression by activating the AMPK/SIRT1/PPARγ pathway (Guan et al., 2010). Chi et al. exposed monocyte-derived macrophages to a combination of BBR and atorvastatin and found that LOX-1 expression decreased progressively with increasing doses of BBR, provided that the atorvastatin dosage was fixed. Furthermore, knockdown of the endothelin-1 (ET-1) receptor prevented the inhibitory effect of the combination of BBR and atorvastatin on LOX-1 expression. Berberine combined with atorvastatin has been suggested to downregulate LOX-1 expression via ET-1 receptors in mono-cytes/macrophages (Chi et al., 2014). In clinical practice, BBR may be therefore combined with statins to enhance their lipid-lowering efficacy.

#### 5.2.2 ABCA1/G1

ABCA1/G1 is an important macrophage receptor that promotes intracellular cholesterol efflux and reduces the formation of macrophage foam cells. The promotion of ABCA1/G1 expression is a promising strategy for AS treatment. BBR increases ABCA1/G1 expression by decreasing the degradation rate of ABCG1 mRNA, whereas the effects of BBR on increasing ABCA1/G1 protein levels and promoting cholesterol efflux are blocked when NRF2 is silenced, suggesting that BBR might exert a protective effect by inhibiting foam formation through the NRF2-mediated ABCA1/G1 signaling pathway (Yang et al., 2020). The regulatory role of LXR in ABCA1/G1 has been reported in many papers (Morin et al., 2020). Knockdown of LXRα mRNA expression by siRNAs abrogates BBR-mediated ABCA1 protein expression and affects macrophage cholesterol efflux. These data suggest that BBR reduces macrophage foam cell formation by enhancing LXRα-ABCA1-dependent cholesterol efflux (Lee et al., 2010). In contrast, another study found that in ox-LDL-induced human macrophage-derived foam cells, BBR treatment has no effect on ABCA1 expression, but rather reduces foam cell formation by inhibiting LOX-1 uptake of ox-LDL (Guan et al., 2010), possibly because of the different culture conditions of the model cells. Further studies are needed to elucidate the effect of BBR on the macrophage cholesterol efflux regulator ABCA1.

### 5.3 BBR inhibits vascular smooth muscle excessive proliferation

The contractile VSMC phenotype is typically observed in healthy vessels. When the vessel is injured, VSMCs are converted to a highly proliferative synthetic phenotype, accelerating VSMC migration and proliferation and the secretion of extracellular matrix molecules, thereby accelerating AS progression (Basatemur et al., 2019). Recent studies have shown that hypercholesterolemia can induce VSMC proliferation through the ERK1/2 (Verdeguer et al., 2007) and Wnt signaling pathways (Zhuang et al., 2016). The role of BBR in preventing VSMC proliferation by reducing circulating lipid levels has been demonstrated in many studies and is closely related to the Wnt (Dong et al., 2022) and ERK1/2 (Qu et al., 2020) pathways. In a murine model of diabetic AS, VSMC proliferation could be induced by hypertension-induced mechanical stretch stress and advanced glycosylation end products (AGEs) alone or in combination. BBR inhibits this progression by decreasing protein disulfide isomerase (PDI) expression to prevent vein graft stenosis (Ping et al., 2017). Endoplasmic reticulum stress (ERS) is essential for VSMC proliferation in CVD (Hetz, 2012). BBR attenuates VSMC proliferation by inhibiting the PDI-ERS system (Wang et al., 2020a). Also, PPARα and NO are involved in the proliferation of VSMCs, and BBR was found to upregulate the PPARα-NO signaling pathway and increase vascular NO production, thereby inhibiting the proliferation of Ang IV-stimulated VSMCs (Qiu et al., 2017). In summary, BBR inhibits the excessive proliferation of VSMC through multiple pathways, thereby maintaining normal vascular function.

## 6 Safety and tolerability of BBR in lipid metabolic diseases

In addition to drug efficacy, drug safety is important for patient health (Tan et al., 2016). There have been reports of adverse effects, such as cardiac discomfort, in some individuals after the use of BBR (Feng et al., 2018a). However, the safety of BBR, a drug that has been used for thousands of years, is well established in clinical practice. In a meta-analysis of the efficacy and safety of BBR in the treatment of dyslipidemia, 16 randomized controlled trials (RCTs) were included, of which 11 reported 164 adverse events (45 in the berberine group and 119 in the control group). Further combined analysis of the incidence of adverse events in each group showed no significant differences (Ju et al., 2018). In addition, non-oral formulations developed to improve the bioavailability of BBR, such as transdermal formulations of BBR and dihydroberberine, showed no changes in renal and hepatic biomarkers during treatment, supporting the safe use of transdermal compositions (Buchanan et al., 2018). Meanwhile, the use of nutraceuticals containing BBR has gradually expanded owing to their better lipid-lowering effects, and no adverse events have been reported during their administration (Riccioni et al., 2018). Statins are routinely used in the treatment of hyperlipidemia, and the incidence of adverse events is not statistically significant when comparing BBR treatment to treatment with statin (Yang et al., 2023).

In addition to adverse drug reactions, tolerability is another concern in clinical practice. Statins are first-line drugs for the treatment of hyperlipidemia. However, even with lower statin doses, intolerance still occurs in about 10%–15% of patients (Banach et al., 2015). Nutritional preparations containing BBR have recently been recommended as alternative lipid-lowering strategies for statin-intolerant patients (Banach et al., 2018). For example, the nutritional combination of red yeast rice, lipotriol, and BBR is effective and well tolerated for lipid management in patients with a low to moderate risk of hypercholesterolemia (Gonnelli et al., 2015). In addition, several clinical trials have comprehensively studied the commercially available nutritional drug Armolipid Plus, which contains red yeast rice, cholesterol, BBR, folic acid, astaxanthin, and coenzyme Q10. Armolipid Plus has been shown to reduce TC and LDL-C levels in patients with mild to moderate dyslipidemia, particularly in those intolerant to statins (Barrios et al., 2017). Therefore, this evidence suggests that berberine-containing nutraceuticals may be a reasonable option for patients with mild to moderate dyslipidemia, particularly those who are intolerant to statins.

## 7 Discussion

Lipid metabolic diseases, such as obesity, hyperlipidemia, NAFLD, and AS, are chronic diseases that require long-term management. Many challenges such as drug resistance and adverse drug reactions occur during drug treatment. BBR is a safe and effective natural product and a potential option for long-term treatment and management of lipid metabolic diseases. Emerging research over the past decade has suggested that BBR may synergistically regulate lipid metabolism in the liver, gut, and blood vessels to combat lipid metabolic diseases, with specific targets involving CD36, MTTP, SR-BI, ACAT, LOX-1, ABCA1, and intestinal flora (Figure 3). Many studies have been conducted on CD36, ACAT, ABCA1, and LOX-1 targets, and there is a clear understanding of the role of these targets and the potential regulatory mechanisms of BBR on them, and thus further high-quality and large-scale studies should be conducted on these targets to facilitate clinical drug development.

However, earlier MTTP-targeting drugs caused side effects. Furthermore, the impact of SR-BI expresson on blood lipids varies between tissues. It is therefore crucial to conduct further research to investigate whether the regulatory effects of BBR on these two targets can effectively reduce blood lipids, while minimizing potential drug side effects. MTTP promotes lipid binding to ApoB and stabilizes ApoB expression, and a lack of MTTP decreases the secretion of ApoB-containing lipoproteins in the gut and liver (Takahashi et al., 2021). Meanwhile, an article published in JAMA on lipids and lipoproteins indicated that lowering ApoB levels was key to successful lipid-lowering therapies compared to LDL-C or TG (Ference et al., 2020); therefore, lowering ApoB levels through the downregulation of MTTP may be a promising lipid-lowering strategy. Lomitapide, a drug that targets the downregulation of MTTP, reduces ApoB secretion but causes hepatic side effects, such as increased liver fat accumulation, which limits its clinical application (Stefanutti, 2020). In contrast, BBR can reverse the abnormal expression of MTTP to alleviate hepatic lipid accumulation (Chen et al., 2021) and may be a potential drug for further lipid lowering in synergy with lomitapide.

SR-BI is expressed in the liver, intestines, and vascular macrophages. However, its function is tissue-specific, with SR-BI on vascular macrophages being responsible for transferring intracellular cholesterol to circulating HDL, thereby reducing macrophage foam cell formation (Yan et al., 2022). Circulating HDL-CE is further taken up by liver SR-BI and converted into BA for excretion into the intestine. When this reverse cholesterol transport functions properly, circulating cholesterol levels are reduced. Although the function of intestinal SR-BI is controversial, one study found that it is present in the intestinal parietal membrane and acts as a high-affinity receptor for intestinal cholesterol (Labonté et al., 2007). However, mice lacking SR-BI exhibit normal or enhanced intestinal cho-lesterol absorption (Mardones et al., 2001). Another study showed that circulating triglyceride-rich lipoproteins (TRL) levels and that of their remnants, cholesterol and ApoB48, were lower in SR-BI−/− mice than in wild-type mice, suggesting that SR-BI may be a novel regulator of CM production (Lino et al., 2015). In Caco-2 cells, ApoB48, a component of AS plaques, is secreted more often when SR-B1 is overexpressed (Fuentes et al., 2019). In rats and Caco-2/TC7 cell lines, LXR reduces CM secretion and alleviates postprandial triglyceridemia by inhibiting SR-B1 via a miRNA post-transcriptional mechanism (Briand et al., 2016). BBR is a known LXR agonist that can improve lipid levels by activating SR-BI in hepatocytes and macrophages, suggesting that BBR may reduce circulating TRL and its remnant levels through intestinal SR-BI (Ma et al., 2021b). Meanwhile, the European Atherosclerosis Society consensus states that TRL and its remnants may contribute significantly to residual cardiovascular risk in patients on optimized LDL-lowering therapy (Ginsberg et al., 2021), suggesting that BBR may be a promising target for the further reduction of residual cardiovascular risk. In addition, activation of LXR in the intestine reduces cholesterol absorption by upregulating intestinal ABCG5/G8 and increases cholesterol efflux by upregulating ABCA1, thus delaying AS progression (Sasso et al., 2010). The modulatory effect of BBR on LXR has been demonstrated in many studies, and BBR may act on intestinal ABCG5/8 to promote intestinal lipid excretion and improve blood lipid levels. Few studies have examined how BBR affects intestinal lipid metabolism targets, and further research in this area should be pursued.

Furthermore, the low solubility and bioavailability of BBR limits its clinical use. However, this is also one of its advantages, as safety is relatively assured, even with rapidly increasing oral doses. Even so, numerous studies have focused on improving the structure of BBR drugs or enhancing their penetration with additives to improve its bioavailability (Imenshahidi and Hosseinzadeh, 2019). Various nanocarriers for encapsulating BBR have small particles, high surface reactivity, and high adsorption capacity, which improve its efficacy and bioavailability (Hori et al., 2023). Mixed micelles loaded with BBR using Pluronic P85 and Tween 80 enhance intestinal absorption and plasma concentrations of BBR by inhibiting P-gp- and CYP450-mediated efflux and metabolism of BBR in the intestine (Kwon et al., 2020). In addition, transdermal formulations of BBR may be more effective in treating dyslipidemia or hypercholesterolemia because of the higher concentration of circulating BBR compared with oral administration (Buchanan et al., 2018).

Thus, BBR is a potential natural compound for use in treating lipid metabolic disorders. Considering the important role of TRL and its remnants in dyslipidemia and atherosclerotic cardiovascular disease processes, and the relatively few studies on the modulation of intestinal lipid metabolic targets by BBR, future in-depth studies in this area may be an important direction for further lipid reduction. Further structural modifications to BBR may improve its oral bioavailability and efficacy. Blends of BBR with other nutraceuticals should also be carefully analyzed, as pure BBR was not used in these studies. Moreover, large-scale, high-quality, and multicenter clinical trials must be carefully designed to assess the safety, toxicological profile, and clinical utility of BBR in patients with lipid metabolism disorders.
