# Correction: Efficacy and underlying mechanisms of berberine against lipid metabolic diseases: a review

**DOI:** 10.3389/fphar.2026.1998036

**Published:** 2026-10-29

**Authors:** Yajie Cai, Qiaoning Yang, Yanqiao Yu, Furong Yang, Ruina Bai, Xiaodi Fan

**Affiliations:** 1 Xiyuan Hospital, China Academy of Chinese Medical Sciences, Beijing, China; 2 National Clinical Research Center for Chinese Medicine Cardiology, Xiyuan Hospital, China Academy of Chinese Medical Sciences, Beijing, China; 3 NMPA Key Laboratory for Clinical Research and Evaluation of Traditional Chinese Medicine, Beijing, China; 4 Department of Graduate School, Beijing University of Chinese Medicine, Beijing, China; 5 Institute of Basic Medical Sciences, Xiyuan Hospital of China Academy of Chinese Medical Sciences, Beijing, China; 6 Key Laboratory of Pharmacology of Chinese Materia Medica, Beijing, China

**Keywords:** berberine, berberine metabolite, intestinal-hepatic-vascular tissue, pharmacological mechanism, lipid metabolic disease

In the published article, there was an error in the characterization of the position of the 2016 European Society of Cardiology/European Atherosclerosis Society Guidelines regarding berberine. The original sentence incorrectly stated that the guidelines recommended berberine as a dietary supplement and functional food for treating dyslipidemia. However, the guidelines summarized evidence suggesting potential lipid-lowering effects of berberine while emphasizing that its efficacy required further assessment in high-quality randomized clinical trials. To provide a balanced description of the available evidence while clearly distinguishing a formal guideline from an expert position paper, the corrected text also cites a 2017 International Lipid Expert Panel position paper that supported the use of BBR as a lipid-lowering nutraceutical in selected populations.

A correction has been made to the section Introduction, paragraph number 2:

The original sentence stated:

“The guidelines of the European Society of Cardiology and the European Atherosclerosis Society recommend BBR as a dietary supplement and functional food for treating dyslipidemia (Members et al., 2016).”

The corrected sentence reads:

“The 2016 ESC/EAS Guidelines summarized evidence suggesting that BBR may reduce low-density lipoprotein cholesterol and triglyceride levels but emphasized that its efficacy for treating dyslipidemia requires further assessment in high-quality randomized clinical trials (Members et al., 2016). Separately, an International Lipid Expert Panel position paper supported the use of BBR as a lipid-lowering nutraceutical, particularly for patients with statin intolerance, mild hypercholesterolemia, or metabolic syndrome (Cicero et al., 2017).”

A new reference has been added accordingly:

Cicero, A. F. G., Colletti, A., Bajraktari, G., Descamps, O., Djuric, D. M., Ezhov, M., et al. (2017). Lipid lowering nutraceuticals in clinical practice: position paper from an International Lipid Expert Panel. Arch. Med. Sci. 13, 965–1005. doi: 10.5114/aoms.2017.69326.

In the published article, five existing references required correction: an incorrect DOI was provided for Xu et al. (2017); the journal title was incorrect for Riccioni et al. (2018); the publication year was incorrect for Cao et al. (2018b); the year designation and article number required correction for Cao et al. (2018a); and the journal title abbreviation was incorrect for Xu and Weng (2020). The five corrected references, together with the corresponding in-text citation changes necessitated by the Cao et al. year correction, are listed below.

The reference for Xu et al. (2017) was erroneously written as:

Xu, R. X., Sun, X. C., Ma, C. Y., Yao, Y. H., Li, X. L., Guo, Y. L., et al. (2017). Impacts of berberine on oxidized LDL-induced proliferation of human umbilical vein endothelial cells. Am. J. Transl. Res. 9, 4375–4389. doi: 10.1016/j.apradiso.2017.05.025.

It should be:

Xu, R. X., Sun, X. C., Ma, C. Y., Yao, Y. H., Li, X. L., Guo, Y. L., et al. (2017). Impacts of berberine on oxidized LDL-induced proliferation of human umbilical vein endothelial cells. Am. J. Transl. Res. 9, 4375–4389.

The reference for Riccioni et al. (2018) was erroneously written as:

Riccioni, G., Gammone, M. A., Currenti, W., and D’Orazio, N. (2018). Effectiveness and safety of dietetic supplementation of a new nutraceutical on lipid profile and serum inflammation biomarkers in hypercholesterolemic patients. Biomacromolecules 23, 1168. doi: 10.3390/molecules23051168.

It should be:

Riccioni, G., Gammone, M. A., Currenti, W., and D’Orazio, N. (2018). Effectiveness and safety of dietetic supplementation of a new nutraceutical on lipid profile and serum inflammation biomarkers in hypercholesterolemic patients. Molecules 23, 1168. doi: 10.3390/molecules23051168.

The reference for Cao et al. (2018b) was erroneously written as:

Cao, S., Xu, P., Yan, J., Liu, H., Liu, L., Cheng, L., et al. (2018b). Berberrubine and its analog, hydroxypropyl-berberrubine, regulate LDLR and PCSK9 expression via the ERK signal pathway to exert cholesterol-lowering effects in human hepatoma HepG2 cells. J. Cell. Biochem. 120, 1340–1349. doi: 10.1002/jcb.27102.

It should be:

Cao, S., Xu, P., Yan, J., Liu, H., Liu, L., Cheng, L., et al. (2019). Berberrubine and its analog, hydroxypropyl-berberrubine, regulate LDLR and PCSK9 expression via the ERK signal pathway to exert cholesterol-lowering effects in human hepatoma HepG2 cells. J. Cell. Biochem. 120, 1340–1349. doi: 10.1002/jcb.27102.

The publication-year correction for Cao et al. also requires the following corresponding changes to the in-text citations and reference-list designation:

In Section 4.4.3 Berberrubine, “Cao et al., 2018b” should read “Cao et al., 2019.”

In Sections 3.2.1 SR-BI, and 3.2.2, LDLR, the two instances of “Cao et al., 2018a” should read “Cao et al., 2018.”

The reference for Cao et al. (2018a) was erroneously written as:

Cao, J., Xu, Y., Li, F., Shang, L., Fan, D., Yu, H., et al. (2018a). Protein markers of dysfunctional HDL in scavenger receptor class B type I deficient mice. J. Transl. Med. 16, 155–213. doi: 10.1186/s12967-018-1502-y.

It should be:

Cao, J., Xu, Y., Li, F., Shang, L., Fan, D., Yu, H., et al. (2018). Protein markers of dysfunctional HDL in scavenger receptor class B type I deficient mice. J. Transl. Med. 16, 155. doi: 10.1186/s12967-018-1502-y.

The reference for Xu and Weng (2020) was erroneously written as:

Xu, S., and Weng, J. (2020). Familial hypercholesterolemia and atherosclerosis: animal models and therapeutic advances. Trends endocrin. Mater. 31, 331–333. doi: 10.1016/j.tem.2020.02.007.

It should be:

Xu, S., and Weng, J. (2020). Familial hypercholesterolemia and atherosclerosis: animal models and therapeutic advances. Trends Endocrinol Metab. 31, 331–333. doi: 10.1016/j.tem.2020.02.007.

The original version of this article has been updated.

